# Transforming primary care: scoping review of research and practice

**DOI:** 10.1108/JICA-03-2018-0023

**Published:** 2018-07-02

**Authors:** Robin Miller, Catherine Weir, Steve Gulati

**Affiliations:** Health Services Management Centre, University of Birmingham, Birmingham, UK

**Keywords:** Integrated care, Primary care, Transformation, Health care home

## Abstract

**Purpose:**

The purpose of this paper is to reflect on research evidence and practice experience of transforming primary care to a more integrated and holistic model.

**Design/methodology/approach:**

It is based on a scoping review which has been guided by primary care stakeholders and synthesises research evidence and practice experience from ten international case studies.

**Findings:**

Adopting an inter-professional, community-orientated and population-based primary care model requires a fundamental transformation of thinking about professional roles, relationships and responsibilities. Team-based approaches can replicate existing power dynamics unless medical clinicians are willing to embrace less authoritarian leadership styles. Engagement of patients and communities is often limited due to a lack of capacity and belief that will make an impact. Internal (relationships, cultures, experience of improvement) and external (incentives, policy intentions, community pressure) contexts can encourage or derail transformation efforts.

**Practical implications:**

Transformation requires a co-ordinated programme that incorporates the following elements – external facilitation of change; developing clinical and non-clinical leaders; learning through training and reflection; engaging community and professional stakeholders; transitional funding; and formative and summative evaluation.

**Originality/value:**

This paper combines research evidence and international practice experience to guide future programmes to transform primary care.

## Introduction

Internationally there is a growing aspiration to rebalance health care systems towards primary rather than secondary care, and for primary care in turn to move towards a population-based model (World Health Organisation, 2015; European Commission, 2017). This reflects common concerns regarding ageing populations, increasing numbers of people living with multiple long-term conditions, negative impacts of lifestyle choices including diet, activity and alcohol consumption, and continuing inequalities within society. There is a recognition that whilst health care services provide a vital contribution, this is only one component of improving the health and wellbeing of communities. Even well-funded health care has a finite capacity and will sit alongside other sources of information, influence and support. Constructive engagement with wider community resources and broader societal institutions is therefore necessary. Peoples’ expectation of the services that they receive or purchase is also changing. Flexibility of access, connection through mobile technology and transparency of information are becoming the norm within other industries and sectors. Personalised health care will require services that are available at evenings and weekends, connected across sectors and settings, and able to communicate through different media. This will in turn require professionals that are comfortable with such new relationships and processes (Frenk *et al.*, 2010; Needham and Mangan, 2014).

New, or at least enhanced, models of primary care are seen as a response to these demographic and societal changes through providing pro-active, holistic and patient-orientated care. They are often designated by terms such as “primary/health care/medical homes” or similar concepts[1]. Despite variation between national contexts, there is remarkable similarity in their core principles – designated populations of about 30-50,000 to provide sufficient scale whilst maintaining connection with communities; inter-professional teams that include the development of new roles to complement traditional disciplines; stratification of need within a population to enable targetted and appropriate responses; and supporting people to take responsibility for their health and access community-based resources. There remains though uncertainty about how best to implement these in practice to ensure genuine and sustained improvements in personal wellbeing, population health and use of resources (Berwick *et al.*, 2008). This paper outlines current knowledge regarding such transformation in primary care from research literature and international practice experience.

## Methodology

The paper is based on a scoping review of primary care transformation (Arksey and O’Malley, 2005; Anderson *et al.*, 2008) undertaken as part of a critical review of an innovation programme. The methodology combines research evidence with international experience of undertaking primary care transformation. The structure of the scoping review is set out below and whilst these are presented in linear order in practice, there was interaction between stages (Dijkers, 2015). Throughout there was engagment with primary care stakeholders to define the questions of interest, seek response to emerging findings and identify other areas for investigation:

1. Clarification of research questions through interviews with primary care stakeholders. This included policy makers, national health bodies, professional associations, and local health oversight boards.
2. Identification of relevant studies through a literature search by a specialist librarian (Box I).
3. Selection of studies that were based on primary research or reviews of research in line with the inclusion/exclusion criteria (Box I). This resulted in 36 articles being included in the final review. To these articles were added notable articles from peer-reviewed literature connected with large-scale change in health and care systems.
  Box I **Overview of literature search**
  Search terms: transformation OR transform OR (transform$ adj3 care AND “primary care” OR “general practice” OR “general practitioners” OR “general practice” OR “primary healthcare” OR “family medicine”
  Databases: HMIC; Social Policy and Practice; CINAHL; Web of Science; ASSIA; Cochrane; SCOPUS; and SCO
  Inclusion: published between 2007 and 2017; English language; based on primary research; and peer reviewed
  Exclusion: commentary rather than research based; not in English; and not focused on primary care transformation
4. Practice experience of undertaking transformation in primary care was gained from those who have led and/or evaluated related projects. A long list of international case examples were identified through the European Forum for Primary Care and the primary care networks of the Health Services Management Centre. The background of potential case studies was gathered through initial discussion via e-mail/or telephone interviews. Final case study selection was based on demonstration of sufficient progress in implementation to provide practical insights of primary care transformation. Further documentation (e.g. strategies, evaluations, published articles) was obtained on each of the case study and between one to three semi-structured interviews completed with people leading and/or evaluating the transformation.
5. Charting of data from the literature and practice experience by a team of three researchers using nVivo software with regular discussion and clarifications of emerging themes.
6. Synthesis of research and practice experience which was presented to primary care stakeholders through a series of three interactive workshops. Stakeholders included clinicians, practitioners, community representatives, managers and policy makers. Following these events further data were gathered as required and synthesised into the final analysis.

This paper is structured around the main themes identified through the analysis with each theme synthesising evidence from research and insights from practical transformation programmes. The case studies of transformation are identified through their title being displayed in italics (Table I).

## Learning from evidence and practice

### Transformation not just improvement

The new model of primary care requires new procedures and accountabilities to ensure that the health, care and other services are appropriately organised and incentivised. Quality improvement, i.e. continuous efforts to improve processes through exploring problems, applying remedies, and monitoring impacts, is also important (Irwin *et al.*, 2015). However, an incremental approach which focuses on technical logistics is not sufficient (Nutting *et al.*, 2009; McGough *et al.*, 2017). A more fundamental redesign is required which entails a new vision of its purpose and contribution to the wellbeing of society (Homer and Baron, 2010; Cronholm *et al.*, 2013). This requires medical clinicians, other professionals and primary care organisations developing new “mental models” or “paradigms” about their professional role. It entails a change in emphasis which puts the interests of people and communities at the centre of their work. New relationships between professionals and their organisations will be required in which there is a willingness to be flexible and adaptive beyond their individual interests and histories (McGough *et al.*, 2017). Therefore, it is a question of “transformation” rather than steady enhancement.

In *Achieving Clinical Excellence*, general practices were given autonomy to deploy the additional funding to respond to the priority needs of their local populations and put in place the enablers for community-based diagnosis and treatment. Despite this being an explicit principle of the programme, it took the general practices several weeks to accept that they had such flexibility. It was only fully recognised following several discussions with the commissioners in the safe environment of a learning set. The *Beacon* initiative found that a major implementation barrier of the enhanced primary care based diabetes service was the perspective of the general practitioners. If they were not in agreement with the new arrangements then they would be unlikely to recommend that patients should access the enhanced primary care service (Jackson *et al.*, 2017). Based on extensive work in North Carolina, three stages of transformation have been suggested – an initiation phase, an intermediate phase, and an advanced phase (Donahue *et al.*, 2013). Each phase has distinct motivators based on internal and external influences. The initiation phase is the process through which practices decide if they are willing to move towards the transformed state. External motivators include professionals bodies, peer comparison and incentive structures. Internal motivators include a wish to “do the right thing”, recognising an opportunity to improve patient care, and becoming more efficient. Having decided to participate not all practices in this programme went on to achieve sustainable transformation (defined as demonstrating substantial improvement in at least three quality domains). Doing so was commonly associated with collective reflection on data, actively including external improvement experts and participating in multi-disciplinary networking.

### Clinical engagement

Primary care involves many more professionals and services than generalist medical clinicians but there is no doubt that they are often at the centre of its organisation and delivery. Their relative autonomy, professional networks and status within society mean that any large-scale change in health care has to consider how to positively engage them in the process (Best *et al.*, 2012). Transformation requires these well-educated professionals to find new ways to communicate and interact with each other as well as with other professionals (Crabtree *et al.*, 2011). This necessitates time and facilitation to reflect on what they do and why they do it. However, the combination of pressured workloads, a culture of autonomy and scepticism on its effectiveness can result in doctors being resistant to investing in such reflection. In *Midlands Health Network*, clinicians were able to spend time away from their clinical practice to think through the change process. Peer groups were an invaluable forum for undertaking this reflection and in sharing learning about how to overcome common challenges. Team-based approaches help to provide additional capacity and more holistic care but can be particularly challenging for doctors (Nutting *et al.*, 2009; Cronholm *et al.*, 2013; Levesque *et al.*, 2017; McGough *et al.*, 2017). This is connected with “deeply held beliefs that PC doctoring was based in a strong, trusting relationship between a patient and a physician” (Russell *et al.*, 2017, p. 23). Changing this traditional arrangement requires doctors to reinterpret their role as contributors to a primary care team. This is helped by demonstrating that their work can be safely diverted to other, educating them about the role and competences of other professionals and providing opportunities to directly engage with these other professionals (McGough *et al.*, 2017).

Team working often requires doctors to develop new skills, and in particular new leadership skills (Nutting *et al.*, 2009; Levesque *et al.*, 2017). Traditionally, they were able to adopt authoritarian approaches in which other professionals were instructed as to the required actions to be undertaken. Instead a team needs to be fostered by more facilitative leadership which encourages the contribution of all members. Medical clinicians owning the practice in which the team operates can lead to their continued domination of inter-professional meetings (Cronholm *et al.*, 2013; Russell *et al.*, 2017). In Ontario, Community Health Centres employ all the clinical staff which has helped to develop more team-based cultures. If other professionals and staff within primary care experience their contributions as being valued then this can level traditional hierarchies and encourage further ideas for transformation (Hilts *et al.*, 2012). In the MacMillan programme, pilot sites were required to identify non-clinical champions as well as clinical champions for change within a practice. This provided an unusual but welcome opportunity for practice managers to lead a clinical transformation.

### Context

Context is widely recognised as an important factor in successfully implementing transformation (Greenhalgh *et al.*, 2009; Health Foundation, 2014). This is equally true in primary care. For example, the Agency for Healthcare Research and Quality provided grants to 14 different projects that had adopted Patient-Centred Medical Homes to enable them to undertake an evaluation of impact and the connected change processes. The review of these evaluations conclude that “the context within which transformation occurred in the practices studied is critical to understanding their success. Contextual factors are diverse and may include both internal and external factors, many of which may be outside the direct control of the practice” (McNellis *et al.*, 2013, p. 54).

Internal factors include a sufficiency of staff and other resources and information systems to support electronic patient records and accurate performance monitoring (Fontaine *et al.*, 2015). Small practices can find it particularly difficult to generate enough capacity to undertake the necessary actions (Goetz Goldberg, 2012; Scholle *et al.*, 2013). Beyond these practical factors, the attitude of staff towards the transformation and internal relationships are central to positive engagement. The process is made more difficult by personality clashes between clinicians and others, an authoritarian leadership style that does not encourage wider engagement, and low-team cohesion (Arar *et al.*, 2011; Hung *et al.*, 2017; Miller-Day *et al.*, 2017). It is easier for a primary care service that is stable, has sufficient resources, experience of quality improvement and good internal relationships to successfully transition to the transformed model. Better implementation is associated with a belief by the staff concerned that the model has value and builds on existing good practice (Wise *et al.*, 2011; McNellis *et al.*, 2013). This increased the likelihood that clinicians and managers will be willing to commit the additional time, be actively looking for opportunities to learn and be ready to accept associated risks. However, if the new approach is seen as being imposed externally and an unnecessary disruption then the opposite is true. This again underlines the conundrum that it is harder to undertake primary care transformation with the services that are in most need of undertaking such change. This was evident in the Beacon programme, in which the practices that had previously been open to hosting external clinicians and other practitioners were comfortable in greater collaboration with the secondary care diabetes specialists. Similarly the inter-professional model adopted by *CASAP* floundered when it was imposed on other practices by the Catalan Health Institute. These practices had not undergone a similar development process and, unlike the staff within *CASAP*, had not chosen to work in such an environment. This contrasts with the approach taken by the National Association of Primary Care in which general practices volunteer to be part of the programme but do not receive any funding as such (Kumpunen *et al.*, 2017).

External factors out with the control of the primary care service also play a major influence. Monetary incentives are not sufficient by themselves to generate transformation but the financial structures in which practices operate can encourage or block more team-based working (Fontaine *et al.*, 2015; Wise *et al.*, 2011; Russell *et al.*, 2017; Wagner *et al.*, 2017). For example, GP Super Clinics were introduced in Australia as part of the National Primary Care Strategy in 2010. They provided purpose-built facilities that could host multiple disciplines which commonly included mental health professionals, community-based nursing, acute specialists and community education providers. The expectation was that co-location would enable the development of shared governance and clinical protocols which in turn would lead to more co-ordinated care. In reality, the continuation of fee-for-service billing meant that clinicians were not able or encouraged to adopt more team-based practice (Lane *et al.*, 2017). The *Macmillan* programme only provided limited funding for participating practices but this was seen as symbolically important and a recognition that they were taking on additional responsibilities. The French Ministry of Health and Social Affairs introduced a payment for team-based working between primary care professionals on top of the existing fee-for-service payments. This accounts for almost 10 per cent of group-based practices income in the *Maison de Sante* and funds the physical estate, management time and multi-disciplinary reviews of individuals with more complex needs.

The political environment, views of professional networks and expectations of the local community influence the readiness of primary care services to consider moving to a new way of working (McNellis *et al.*, 2013; Russell *et al.*, 2017). This relates to both their sense of what is expected, and confidence that the risks associated with new and unfamiliar territory will gain wider support. *Wellbeing Enterprises* involved the commissioning of a social enterprise to introduce new opportunities for patients to access local resources. This enabled primary care services to draw on a wider range of community assets than was previously the case and opened up opportunities to draw on additional charitable grants and other income sources (Swift, 2017). Facilitation that is external to the primary care service can provide additional insights, capacity and objectivity (Lane *et al.*, 2017). The MacMillan programme recruited three external facilitators to support practices with the practical changes connected with implementing the new cancer pathway. This support was universally appreciated as it provided additional capacity, expertise and objectivity. An inter-disciplinary change management team provided similar support practices adopting the medical home model in the *Midlands Health Network* programme.

### Patient and community engagement

Putting people and their communities at the centre is one of the core principles of the models of transformed primary care. Despite this, the evidence suggests that many practices find it difficult to reflect this principle in reality. For example, one study of smaller practices (i.e. less than five doctors) adopting the medical home model reported that whilst 30 per cent said they had trained clinicians and staff on involving patients or consumer advocates, only 15 per cent of practices actively included patients on quality improvement teams (Scholle *et al.*, 2013). The authors suggest that stronger evidence is needed of the positive impacts which will result from such engagement to convince practices that it is worth the effort. However, research evidence is not that well developed, with one recent review concluding that “there is a paucity of published research on patient engagement at the practice level in general and in the primary care setting in particular, with very little of the research that has been conducted consisting of rigorous, controlled studies investigating triple aim outcomes” (Sharma and Grumbach, 2017, p. 264) The lack of formal evidence does not mean that there is no impact – rather that there is an insufficient number of rigorous research supported by valid and reliable tools. In Minnesota for practices to receive accreditation as a Health Care Home required demonstration of patient participation (Fontaine *et al.*, 2015). This encouraged innovative ways to enable patients to be engaged such as patient advisory councils and training of “patient partners”. Previously only 32 per cent of these practices regularly provided opportunities for patients to be actively engaged but following the mandate this became 100 per cent. This was connected with interviewees in the study with their own “personal satisfaction and career-renewing energy”. The *House of Care* pilot sites which invested funding to develop or enhance a patient engagement infrastructure were much more successful in this regard. This was achieved through employing a new member of staff or commissioning an external organisation with skills in this area. In Ontario, the *South Riverdale Community Health Centre* board is comprised of members of the local community (i.e. those who share the values, live in the catchment area and receive a service) with applications being encouraged from populations who are not currently represented. This followed a previous challenge that the Centre was not sufficiently engaging with local people that resulted in a major refocusing of the culture of the service.

Alongside engagement with the process of transformation is a need for patients to be given support and opportunity to be more engaged and accountable for their own health and wellbeing. This can require a different set of behaviours for some patients and a fundamental change of their identity as a patient (McGough *et al.*, 2017). This means that people’s knowledge, skills and confidence to be an active patient have to be considered. Where they are lacking the necessary competences then training and other support will be required. The inter-professional team will have an on-going role to continually build up the ability and confidence of patients. *The House of Care* programmes places considerable emphasis on enabling people to be prepared and informed to engage in collaborative care and support planning. *Wellbeing Enterprises* employed Community Wellbeing Officers who develop a personalised plan for wellbeing with patients. This incorporates how they can incorporate new behaviours in their daily living and facilitating access to other community resources (Swift, 2017).

### Redistribution of resources

The strengthening of primary care is seen as a vehicle not only to improve quality and address inequalities but also to deploy the available resources more efficiently. This is largely based on the assumption that enhanced primary care which is more accessible and responsive to people’s needs will lead to reduction in overall activity by health care providers and a diversion of activity from acute to community settings. Despite this being a common expectation, the evidence to support the redeployment of resources and connected financial savings is not always convincing. For example, a review of 27 initiatives to shift the balance of care reports that whilst there is evidence that some can lead to cost savings many did not, and some had led to increased costs (Imison *et al.*, 2017). It concludes that estimated level of savings are often unrealistic due to unforeseen difficulties in removing fixed costs or a failure to take into account the full resources required to introduce a new intervention. Furthermore ensuring there is sufficient capacity in primary care will be essential for most of these approaches to be successful. One of the reasons that it can be hard to release savings is that health care planners in public health systems find it difficult to disinvest in existing services. Challenges include inconclusive evidence, community resistance and disincentives for clinicians (Williams *et al.*, 2017).

Other evidence reviews have come to a similar conclusion that whilst enhanced models of primary and integrated care have the potential to reduce hospital activity, they struggle to do so at the level which can result in major and sustainable savings (Martínez-González *et al.*, 2014; Van den Heede and Van de Voorde, 2016; Damery *et al.*, 2016). In addition to this formal research evidence are case examples in which health care regions report reducing hospital activity through taking a more systems-based approach (Gottlieb, 2013; Staines *et al.*, 2015; Schluter *et al.*, 2016). Whilst often not of a standard that would be incorporated within a systematic review these examples do still at least suggest that it is possible to have a significant influence on previous levels of investment and the quality of a health care system. This requires sustained efforts over long time periods with continuity in senior leadership being a common feature. Starting from a low base, i.e. particularly fragmented relationships and little experience of introducing innovations makes adoption difficult but also leads to more noticeable levels of improvement than areas in which there has already been progress.

### Programmes not interventions

A common finding from evidence is that programmes of integration are more likely to lead to a rebalancing of resources from acute to primary care and enabling a more pro-active and less crisis-orientated system (Damery *et al.*, 2016; McLellan, 2017; Miller, 2017). Similarly, primary care transformation requires a co-ordinated programme rather than emphasis of one intervention in particular. Building on the experiences of the case studies, it would appear that there are six elements that are commonly incorporated (Table II):

1. external facilitation to provide additional capacity and expertise in undertaking transformation;
2. supporting the development of local clinical and non-clinical leaders;
3. on-going learning in relation to the development of new skills and reflection on emerging evidence of process and impact;
4. stakeholder engagement, in particular patients, communities and wider clinical networks, through sufficient investment in associated infrastructure;
5. transitional funding to enable continuation of existing activities whilst new approaches are introduced; and
6. robust evaluation which provides formative and summative insights against clear objectives and baselines.

There is a need for such programmes to both be endorsed by senior leaders and provide opportunity for those on the frontline to introduce innovations, i.e. “designated” (senior) and “distributed” (those closer to the frontline) leadership (Best *et al.*, 2012; Perla *et al.*, 2013). Establishing forums which support senior-level decision making can be an efficient means to address potential organisational barriers (Starling, 2017). Similarly within frontline services organising opportunities for all associated staff, not just medical clinicians, to share their perspectives can lead to greater engagement and creative solutions being identified (Hung *et al.*, 2017; Starling, 2017).

### Use of data

Programmes also need to be informed by relevant data at the individual, team, clinic and organisation levels. This should include data that reflect the quadruple aim, including clinical performance, patient satisfaction survey and levels of stress experienced by staff (McNellis *et al.*, 2013; McGough *et al.*, 2017). Willingness to actively use such data to drive decision making and influence how a primary care service works is a core behaviour of transformed practices (McNellis *et al.*, 2013; McGough *et al.*, 2017). An “active review of the practice’s performance appeared to help reinforce the value of PCMH, since identifying the gaps in care motivated the teams to work on improving it” (Wise *et al.*, 2011, p. 416). The converse is also true. Practices in which the new model was less well implemented would be much more passive in obtaining and reviewing data (Wise *et al.*, 2011). Data are seen as a crucial element in the *Midlands Health Network* programme as evidence of positive impact that helps to maintain momentum whilst also identifying further areas for improvement. The ease with which data can be generated, collected and understood is important. It must also be seen as relevant and timely if it is going have influence on wider stakeholders (Greenhalgh *et al.*, 2009).

## Conclusion

Many of the components of the new model of primary care are already in existence in most countries – a generalist specialism within medicine, community-based nursing, therapy services and pharmacy services, voluntary organisations responding to different social needs, and public and/or independent organisations which can provide short and long-term domiciliary support. Bringing these together into more integrated and holistic models will require significant reframing by professionals and practitioners and the organisations that they own or employed. This reframing relates to their role, their relationship with others and the resources for which they have lead or sole responsibility. Accountability to and involvement of patients, families and communities will also need a radical overhaul to ensure that people are truly put at the centre of the vision and associated delivery. This scoping review suggests that whilst challenging it is possible to achieve sustainable transformation with a supportive political and social context, and a co-ordinated programme of change. Finally, it could also be argued that whilst these new models of primary care are undoubtedly a step forward they are still limited. Other aspects of primary care such as dentistry and social work are rarely included to any substantial degree, and many of them still expect that delivery will be mainly through medically owned organisations. It will be important therefore that we do not see such models as the end point, but rather another stepping stone on the transformation to more integrated care.

## Figures and Tables

**Table I:** International case examples

Case study	Locality	Overview
Pinnacle Midlands Health Network	New Zealand	Introduction of holistic model within general practice through co-ordination, new roles, technology, and access centre
Achieving Clinical Excellence Programme	England	Clinically led pilots seeking to achieve more holistic care across primary, secondary and social care
Wellbeing Enterprises	England	Asset-based working through person centred reviews led by social enterprise
Shared Care for Diabetes (Beacon)	Australia	Acute – primary care collaboration to enable more community-based care for patients with diabetes
Consorci Castelldefels Agents Salut (CASAP)	Spain	Increasing roles of primary care nurses and reception staff to enable more team-based care
Maison de Sante	France	Inter-professional team working in primary care in shared premises
National Association of Primary Care Medical Home Programme	England	Integrated health and social care model seeking to both personalise care and improve population level outcomes
British Heart Foundation House of Care	Scotland and England	Patient led model of holistic primary care for those with long-term conditions
Ontario Community Health Centres	Canada	Inter-professional team governed through community engagement
MacMillan Cancer Improvement Partnership	England	Strengthening primary care to enable more holistic and primary care orientated cancer pathway

**Table II:** Common transformation programme elements

Element	Pinnacle Midlands Health Network	Achieving Clinical Excellence	British Heart Foundation House of Care	MacMillan Cancer Improvement Partnership
External facilitation	A multi-disciplinary change management team included GPs, managers, analysts and patient partners	One pilot funded an expert in improvement methodology to guide their approach	External support was provided through the Year of Care Partnership and the Health and Social Care Alliance Scotland. Funding was used to employ project managers	Three change facilitators with nursing / primary care backgrounds were recruited to support each practice
Leadership support	Peer leadership networks and time for reflection was provided for GPs, nurses and practice manager leads	Pilot leads had time for reflection and peer challenge within learning sets	Each site identified local clinical leads to champion the new approach	MacMillan GPs helped develop standards and each practice needed a clinical and non-clinical cancer champion
On-going learning	Leadership development programmes were available	Evaluation provided emerging insights to help shape further implementation.	The Year of Care Partnership delivered training on care and support planning and also developed local trainers	Opportunity for leads to meet and reflect on progress with implementation
Stakeholder engagement	Patient forums and multi-disciplinary workshops are held	Sharing events were held for wider GP membership	Patient representatives required on national and local steering groups	A patient engagement facilitator co-ordinated various engagement opportunities
Transitional funding	One-off funding to provide backfill and meet additional expenses	Pilots received additional per capita funding for 12 months with flexibility as to how this was used	Additional funding was provided for project management/patient engagement but not backfill	Practices were offered one-off incentive payments
Robust evaluation	Progress reviewed regularly against baseline	An external formative evaluation was commissioned	An external evaluation was commissioned which also supported local evaluations by sites	An external evaluation was commissioned

## Note

1. Sample examples of population based primary care models include – www.pcpcc.org/about/medical-home; http://napc.co.uk/primary-care-home/; www.health.gov.au/internet/main/publishing.nsf/Content/health-care-homes; and www.aohc.org/model-health-and-wellbeing
